# Supplementary material for: Conversational artificial intelligence HeAlth supporT in Atrial Fibrillation Self-Management (CHAT-AF-S): rationale and randomised controlled trial design
Source: BMJ Open. 2026 Aug 7;16(8):e120834. doi: 10.1136/bmjopen-2026-120834 (PMC13475244; doi:10.1136/bmjopen-2026-120834)
Supplement: Supplementary file 1 [file bmjopen-16-8-s001.pdf]

Supplementary material

Conversational HeAlth supporT in Atrial Fibrillation Self-Management (CHAT-AF-S): a protocol for a randomised controlled trial involving conversational AI

Supplement 1. SPIRIT-AI Checklist - Recommended items to address in a protocol and related documents for clinical trials evaluating AI interventions[1] 2

Supplement 2. Mobile health (mHealth) evidence reporting and assessment (mERA) checklist[2] 5

Supplement 3. Participant Information and Consent Form 7

Supplement 4. Information letter for General Practitioner 17

Supplement 5. Central Monitoring Unit 18

Supplement 6. CHAT-AF-S behaviour change features 20

Supplement 7. Study-specific questionnaire 21

References 23

Supplement 1. SPIRIT-AI Checklist - Recommended items to address in a protocol and related documents for clinical trials evaluating AI interventions[1]

Section		SPIRIT AI Item	Addressed on Page No [b]
Title	1	Descriptive title identifying the study design, population, interventions, and, if applicable, trial acronym	1
		Indicate that the intervention involves artificial intelligence / machine learning and specify the type of model. Specify the intended use of the AI intervention.	1
Trial registration	2a	Trial identifier and registry name. If not yet registered, name of intended registry	3
	2b	All items from the World Health Organization Trial Registration Data Set	N/A
Protocol version	3	Date and version identifier	N/A
Funding	4	Sources and types of financial, material, and other support	23
Roles and responsibilities	5a	Names, affiliations, and roles of protocol contributors	23
	5b	Name and contact information for the trial sponsor	23
	5c	Role of study sponsor and funders, if any, in study design; collection, management, analysis, and interpretation of data; writing of the report; and the decision to submit the report for publication, including whether they will have ultimate authority over any of these activities	23
	5d	Composition, roles, and responsibilities of the coordinating centre, steering committee, endpoint adjudication committee, data management team, and other individuals or groups overseeing the trial, if applicable (see Item 21a for data monitoring committee)	8
Background and rationale	6a	Description of research question and justification for undertaking the trial, including summary of relevant studies (published and unpublished) examining benefits and harms for each intervention	5-6
		Explain the intended use of the AI intervention in the context of the clinical pathway, including its purpose and its intended users (e.g. healthcare professionals, patients, public). Describe any pre-existing evidence for the AI intervention	5-6
	6b	Explanation for choice of comparators	7
Objectives	7	Specific objectives or hypotheses	6
Trial design	8	Description of trial design including type of trial (eg, parallel group, crossover, factorial, single group), allocation ratio, and framework (eg, superiority, equivalence, noninferiority, exploratory)	7
Study setting	9	Description of study settings (eg, community clinic, academic hospital) and list of countries where data will be collected. Reference to where list of study sites can be obtained. Describe the onsite and offsite requirements needed to integrate the AI intervention into the trial setting	7
Eligibility criteria	10	Inclusion and exclusion criteria for participants. If applicable, eligibility criteria for study centres and individuals who will perform the interventions (eg, surgeons, psychotherapists)	8
		State the inclusion and exclusion criteria at the level of participants. State the inclusion and exclusion criteria at the level of the input data.	8
Interventions	11a	Interventions for each group with sufficient detail to allow replication, including how and when they will be administered	8-12
		State which version of the AI algorithm will be used.	8-12
		Specify the procedure for acquiring and selecting the input data for the AI intervention.	8-12
			8-12

		Specify the procedure for assessing and handling poor quality or unavailable input data.<br><br>Specify whether there is human-AI interaction in the handling of the input data, and what level of expertise is required for users<br><br>Specify the output of the AI intervention<br><br>Explain the procedure for how the AI intervention's output will contribute to decision-making or other elements of clinical practice.	
	11b	Criteria for discontinuing or modifying allocated interventions for a given trial participant (eg, drug dose change in response to harms, participant request, or improving/worsening disease)	8-12
	11c	Strategies to improve adherence to intervention protocols, and any procedures for monitoring adherence (eg, drug tablet return, laboratory tests)	8-12
	11d	Relevant concomitant care and interventions that are permitted or prohibited during the trial	8-12
Outcomes	12	Primary, secondary, and other outcomes, including the specific measurement variable (eg, systolic blood pressure), analysis metric (eg, change from baseline, final value, time to event), method of aggregation (eg, median, proportion), and time point for each outcome. Explanation of the clinical relevance of chosen efficacy and harm outcomes is strongly recommended	12-13
Participant timeline	13	Time schedule of enrolment, interventions (including any run-ins and washouts), assessments, and visits for participants. A schematic diagram is highly recommended (see Figure)	7
Sample size	14	Estimated number of participants needed to achieve study objectives and how it was determined, including clinical and statistical assumptions supporting any sample size calculations	14
Recruitment	15	Strategies for achieving adequate participant enrolment to reach target sample size	8
Sequence generation	16A	Method of generating the allocation sequence (eg, computer-generated random numbers), and list of any factors for stratification. To reduce predictability of a random sequence, details of any planned restriction (eg, blocking) should be provided in a separate document that is unavailable to those who enrol participants or assign interventions	13-14
Allocation concealment mechanism	16b	Mechanism of implementing the allocation sequence (eg, central telephone; sequentially numbered, opaque, sealed envelopes), describing any steps to conceal the sequence until interventions are assigned	13-14
Implementation	16c	Who will generate the allocation sequence, who will enrol participants, and who will assign participants to interventions	13-14
Blinding (masking)	17a	Who will be blinded after assignment to interventions (eg, trial participants, care providers, outcome assessors, data analysts), and how	13-14
	17b	If blinded, circumstances under which unblinding is permissible, and procedure for revealing a participant's allocated intervention during the trial	13-14
Data collection methods	18a	Plans for assessment and collection of outcome, baseline, and other trial data, including any related processes to promote data quality (eg, duplicate measurements, training of assessors) and a description of study instruments (eg, questionnaires, laboratory tests) along with their reliability and validity, if known. Reference to where data collection forms can be found, if not in the protocol	13
	18b	Plans to promote participant retention and complete follow-up, including list of any outcome data to be collected for participants who discontinue or deviate from intervention protocols	14
Data management	19	Plans for data entry, coding, security, and storage, including any related processes to promote data quality (eg, double data entry; range checks for data values). Reference to where details of data management procedures can be found, if not in the protocol	15
Statistical methods	20a	Statistical methods for analysing primary and secondary outcomes. Reference to where other details of the statistical analysis plan can be found, if not in the protocol	14-15
	20b	Methods for any additional analyses (eg, subgroup and adjusted analyses)	Will be reported in statistical analysis plan, which will be published in OSF before dataset unlock.
	20c	Definition of analysis population relating to protocol non-adherence (eg, as randomised analysis), and any statistical methods to handle missing data (eg, multiple imputation)	

Data monitoring	21a	Composition of data monitoring committee (DMC); summary of its role and reporting structure; statement of whether it is independent from the sponsor and competing interests; and reference to where further details about its charter can be found, if not in the protocol. Alternatively, an explanation of why a DMC is not needed	15
	21b	Description of any interim analyses and stopping guidelines, including who will have access to these interim results and make the final decision to terminate the trial	N/A
Harms	22	Plans for collecting, assessing, reporting, and managing solicited and spontaneously reported adverse events and other unintended effects of trial interventions or trial conduct<br><br>Specify any plans to identify and analyse performance errors. If there are no plans for this, explain why not.	Adverse events and serious adverse events will be recorded, with indication of whether they are likely related to the study or not. Performance errors of the AI intervention will be analysed as part of the process evaluation.
Auditing	23	Frequency and procedures for auditing trial conduct, if any, and whether the process will be independent from investigators and the sponsor	15
Research ethics approval	24	Plans for seeking research ethics committee/institutional review board (REC/IRB) approval	15
Protocol amendments	25	Plans for communicating important protocol modifications (eg, changes to eligibility criteria, outcomes, analyses) to relevant parties (eg, investigators, REC/IRBs, trial participants, trial registries, journals, regulators)	Protocol amendments will be approved by the Ethics Committee and updated in the trial registration.
Consent or ascent	26a	Who will obtain informed consent or assent from potential trial participants or authorised surrogates, and how (see Item 32)	15
	26b	Additional consent provisions for collection and use of participant data and biological specimens in ancillary studies, if applicable	N/A
Confidentiality	27	How personal information about potential and enrolled participants will be collected, shared, and maintained in order to protect confidentiality before, during, and after the trial	15
Declaration of interests	28	Financial and other competing interests for principal investigators for the overall trial and each study site	2
Access to data	29	Statement of who will have access to the final trial dataset, and disclosure of contractual agreements that limit such access for investigators	14
Ancillary and post-trial care	30	Provisions, if any, for ancillary and post-trial care, and for compensation to those who suffer harm from trial participation<br><br>State whether and how the AI intervention and/or its code can be accessed, including any restrictions to access or re-use.	AI technology is proprietary and provided by Nuance. Intervention and study materials will be freely available in Open Science Framework.
Dissemination policy	31a	Plans for investigators and sponsor to communicate trial results to participants, healthcare professionals, the public, and other relevant groups (eg, via publication, reporting in results databases, or other data sharing arrangements), including any publication restrictions	15-16
	31b	Authorship eligibility guidelines and any intended use of professional writers	We will follow ICMJE guidance
	31c	Plans, if any, for granting public access to the full protocol, participant-level dataset, and statistical code	16
Informed consent materials	32	Model consent form and other related documentation given to participants and authorised surrogates	15
Biological specimens	33	Plans for collection, laboratory evaluation, and storage of biological specimens for genetic or molecular analysis in the current trial and for future use in ancillary studies, if applicable	N/A

[a] It is strongly recommended that this checklist be read in conjunction with the SPIRIT 2013 Explanation & Elaboration for important clarification on the items; [b] Indicates page numbers to be completed by authors during protocol development.

Rivera SC, Liu X, Chan A-W, Denniston AK, Calvert MJ, Ashrafian H, et al. Guidelines for clinical trial protocols for interventions involving artificial intelligence: the SPIRIT-AI extension. The Lancet Digital Health 2020;2(10):e549-e560.

Supplement 2. Mobile health (mHealth) evidence reporting and assessment (mERA) checklist[2]

Criteria	Item no	Notes	Section/Page
**Infrastructure (population level)**	1	Clearly presents the availability of infrastructure to support technology operations in the study location. This refers to physical infrastructure such as electricity, access to power, connectivity etc. in the local context.	8-12
**Technology platform**	2	Describes and provides justification for the technology architecture. This includes a description of software and hardware and details of any modifications made to publicly available software	8-12
**Interoperability/ Health information systems (HIS) context**	3	Describes how mHealth intervention can integrate into existing health information systems. Refers to whether the potential of technical and structural integration into existing HIS or programme has been described irrespective of whether such integration has been achieved by the existing system	The platform is not integrated into existing health information systems.
**Intervention delivery**	4	The delivery of the mHealth intervention is clearly described. This should include frequency of mobile communication, mode of delivery of intervention (that is, SMS, face to face, interactive voice response), timing and duration over which delivery occurred	8-12
**Intervention content**	5	Details of the content of the intervention are described. Source and any modifications of the intervention content is described	8-12
**Usability/content testing**	6	Describe formative research and/or content and/or usability testing with target group(s) clearly identified, as appropriate	8-12
**User feedback**	7	Describes user feedback about the intervention or user satisfaction with the intervention. User feedback could include user opinions about content or user interface, their perceptions about usability, access, connectivity, etc	8-12
**Access of individual participants**	8	Mentions barriers or facilitators to the adoption of the intervention among study participants. Relates to individual-level structural, economic and social barriers or facilitators to access such as affordability, and other factors that may limit a user's ability to adopt the intervention	8-12
**Cost assessment**	9	Presents basic costs assessment of the mHealth intervention from varying perspectives. This criterion broadly refers to the reporting of some cost considerations for the mHealth intervention in lieu of a full economic analysis. If a formal economic evaluation has been undertaken, it should be mentioned with appropriate references. Separate reporting criterion are available to guide economic reporting	To be conducted
**Adoption inputs/ programme entry**	10	Describes how people are informed about the programme including training, if relevant. Includes description of promotional activities and/or training required to implement the mHealth solution among the user population of interest	8-12

**Limitations for delivery at scale**	11	Clearly presents mHealth solution limitations for delivery at scale	8-12
**Contextual adaptability**	12	Describes the adaptation, or not, of the solution to a different language, different population or context. Any tailoring or modification of the intervention that resulted from pilot testing/usability assessment is described	8-12
**Replicability**	13	Detailed intervention to support replicability. Clearly presents the source code/screenshots/ flowcharts of the algorithms or examples of messages to support replicability of the mHealth solution in another setting	8-12
**Data security**	14	Describes the data security procedures/ confidentiality protocols	8-12, 15
**Compliance with national guidelines or regulatory statutes**	15	Mechanism used to assure that content or other guidance/information provided by the intervention is in alignment with existing national/regulatory guidelines and is described	15-16
**Fidelity of the intervention**	16	Was the intervention delivered as planned? Describe the strategies employed to assess the fidelity of the intervention. This may include assessment of participant engagement, use of backend data to track message delivery and other technological challenges in the delivery of the intervention	To be conducted as part of process evaluation

Agarwal S, LeFevre AE, Lee J, L'engle K, Mehl G, Sinha C, et al. Guidelines for reporting of health interventions using mobile phones: mobile health (mHealth) evidence reporting and assessment (mERA) checklist. bmj 2016;352.

Supplement 3. Participant Information and Consent Form

[Insert Institutional logo]

Participant Information Sheet/Consent Form

Interventional Study - *Adult providing own consent*

[site]

**Title**	Conversational HeAlth supporT in Atrial Fibrillation Self-Management
**Short Title**	CHAT-AF-S
**Coordinating Principal Investigator/ Principal Investigator**	Professor Clara Chow
**Location**	**[site]**

Part 1 What does my participation involve?

1 Introduction

You are invited to take part in this research project. The project aims to support people diagnosed with atrial fibrillation who receive either cardiology inpatient or outpatient services at **[site]**. Researchers want to give support and improve self-management and quality of life of people living with atrial fibrillation.

The study is an artificial intelligence-supported (AI-supported) intervention that will involve a series of automated phone calls and text messages delivered over a 3-month period. We will ask you to complete surveys at baseline, 3 months after recruitment, to help us determine the impact of the intervention on AF-related quality of life, knowledge, confidence in self-management of AF, AF self-management behaviours, and health care service utilisation.

This Participant Information Sheet/Consent Form tells you about the research project. It explains what is involved. Knowing what is involved will help you decide if you want to take part in the research.

Please read this information carefully. Ask questions about anything that you don't understand or want to know more about. Before deciding whether or not to take part, you might want to talk about it with a relative, friend or your local doctor.

Participation in this research is voluntary. If you don't wish to take part, you don't have to. You will receive the best possible care whether or not you take part.

If you decide you want to take part in the research project, you will be asked to sign the consent section.

By signing it you are telling us that you:
• Understand what you have read
• Consent to take part in the research project
• Consent to have the tests and treatments that are described
• Consent to the use of your personal and health information as described.

You will be given a copy of this Participant Information and Consent Form to keep.

2 What is the purpose of this research?

The aim of this study is to evaluate the effectiveness of an AI-supported intervention that comprises automated phone calls and text messages to improve self-management and quality of life of people with atrial fibrillation.

Atrial fibrillation (AF) is the most frequently encountered arrythmia globally. It is an increasing burden for health care systems and a major contributor to stroke. It is essential that AF is managed with rate and rhythm control, anticoagulation, and risk factor management to prevent stroke. A large part of AF management is enacted by patients in their daily lives. Digital tools can facilitate patient self-management by assisting with monitoring of physiologic parameters, providing reminders for medication adherence, and supporting other self-care tasks.

This research has been initiated by the study doctor, Professor Clara Chow, a Cardiologist at Westmead Hospital and Academic Director of Westmead Applied Research Centre (WARC).

This research has been funded by Digital Health CRC Limited ("DHCRC"). DHCRC is funded under the Commonwealth's Cooperative Research Centres (CRC) Program.

This research is being conducted by the Westmead Applied Research Centre (WARC) and supported by the Digital Health CRC Limited. The WARC is a research centre at the University of Sydney and located at Westmead Hospital. The DHCRC is funded under the Commonwealth's Cooperative Research Centres (CRC) Program that aims to improve health engagement through the use of digital technology. The company providing the technology for this project is called Nuance Communications. The questions and information that you will receive was developed by talking with people living with AF, and heart doctors with experience in atrial fibrillation.

3 What does participation in this research involve?

People will be eligible to participate if they (1) are >18 years old, (2) have a documented diagnosis of AF, (3) have a mobile phone that is able to receive calls, (4) are able to receive text messages and open weblinks embedded in them, and (5) are competent with English, as ascertained by the study researcher. Participants will be excluded from the study if they (1) are on concurrent active intervention studies focused on atrial fibrillation education and self-management (2) have a concomitant illness, physical impairment or mental condition which in the opinion of the study team/primary care physician could interfere with the conduct of the study including outcome assessment (e.g. hearing impairment not corrected by a digital hearing aid), (3) are pregnant, (4) have a medical illness with anticipated life expectancy of < 3 years, and (5) are unable or unwilling to provide written consent.

If you agree to take part in the study, we ask that you sign and date the consent form. Your participation will be for 3 months. The total study duration (including recruitment and analysis) is two years.

This is a randomised controlled research project which means we put people into groups and use different methods with each group. The results are then compared to see if one is better than the other. Each participant is put into a group by chance (randomly), and you have an equal chance of being placed in each group (1:1 ratio). This research project has been designed to make sure the researchers interpret the results in a fair and appropriate way and avoids study doctors or participants jumping to conclusions. Every participant will continue to receive their usual care no matter which group they are placed in.

The two groups are called 1) Control and 2) Intervention.

1) **Control group: Usual care and completion of surveys**

Participants placed in the "control" group will continue to receive usual care, and only be asked to complete a digital survey at the beginning of the research project and again at 3 months but will not receive regular contact from the research team. The results from this group will be compared to the "intervention" group.

2) **Intervention group: Digital support program**

Participants in the "intervention" group will receive a series of six automated phone calls (two weeks apart), which will collect your answers to certain questions over the phone. The voice on the phone call is a recorded voice (not a live person) but can understand your answers and reply based on the answers you give. These phone calls will be initiated within 30 days post discharge from hospital services. These answers will be sent back to the research team. You will also receive text messages containing internet links to fact sheets, videos or other information that has been approved by heart doctors. This information will help you manage your atrial fibrillation day-to-day. We will also ask you to complete surveys at the beginning of the research project, and again after 3 months.

Data rates may apply when opening internet links. Otherwise, there are no additional costs associated with participating in this research project, nor will you be paid. All aspects of the research project will be provided to you free of charge.

Semi-structured interviews

At the end of the intervention, some participants from the intervention group will be invited to participate in a semi-structured interview. A diverse range of participants will be purposefully selected to obtain a well-round view on the feasibility of the intervention. We aim to include ethnically, culturally and socio-economically diverse individuals, from a variety of age, education level and ethnic groups. Selected participants will be contacted via phone. The interview will be conducted virtually at a time mutually convenient to the participant and the researcher. We will assess the impact of the intervention on the participant journey and care integration, barriers and enablers to implementation, and acceptability and engagement with the intervention.

Interviews will be conducted until data saturation is reached (we predict a minimum of 20 participants will be interviewed and estimate that each interview will last between 10 and 30 minutes).

We will audio-record all interviews with participants for the purpose of transcribing the interview and subsequent analysis of the data. Interview transcriptions will be performed in-house by a research assistant on the project or conducted by a professional transcription service after a confidentiality agreement has been signed. Participants will be notified in advance and asked to consent to the audio recording and will be advised that the recording will be transcribed. Transcripts will be imported into Nvivo version 11.0 (QSR International), a program for managing and analysing qualitative data for thematic analysis. Initial open coding of transcripts will be undertaken by the research team in accordance with the evaluation questions and study objectives. Initial themes will be identified and discussed by the study investigators. Further open coding will be used to determine additional factors in local planning in accordance with the evaluation questions and study objectives.

Information we collect

If you agree to participate, we will use your first name and mobile phone number to make the phone calls and send text messages personal to you. This will be recorded in a database program called REDCap. When entering your information into the database, you will be given a study ID for example '001'. This is to make sure your identity is protected.

We will also collect some information about your relevant medical history from your electronic health records at the hospital. This information will be protected and remain confidential, also using a study ID, removing all identifying information and stored securely at the University of Sydney. The information we collect will help us understand if the CHAT-AF-S program is useful or not.

This research project has been designed to ensure the researchers interpret the results in a fair and appropriate way.

This study will be conducted and monitored by researchers at the Westmead Applied Research Centre (WARC), University of Sydney.

4 What do I have to do?

The following outlines the study activities:

a) You will be required to provide written consent or verbal consent over the phone to be enrolled in this study. Participants who are physically at **[site]** at the time of recruitment will be able to provide written consent. Participants who consent over the phone or via e-consent will be sent a copy via email.

b) Once you have consented, you will be asked some questions about your current quality of life as a person living with AF and what kind of symptoms you are experiencing. We will also collect your contact information like your first name, phone number to ensure we can contact you for this project.

c) You will enter one of 2 study groups, by chance. Each group will receive one of 2 possible treatment options that can include phone calls and text messages for a duration of 3 months. Participants in the intervention group will receive a total of six automated phone calls, occurring 2 weeks apart . The intervention will be initiated within 30 days post discharge from hospital services.

d) Depending on which group you are allocated to, you may be invited to participate in a semi-structured interview. The interviews will be audio-recorded and conducted virtually at a time that is mutually convenient to the participant and the researcher. A designated researcher on the project team will be facilitating the interview. The interviews will help us evaluate the impact and acceptability of this automated communication program

e) For the 3 months you are enrolled in this study, information from your hospital visits will be collected, including medical history and medications. This information will only be accessible by the research team, information is shared only with researchers approved by the lead researcher on the project and is kept secure and confidential. This information will help the research team understand the different kinds of patient experiences.

f) During the course of the study the researchers will ask participants to complete up to six different surveys. These surveys will help the research team track how participants are feeling over time. The surveys are listed below and the approximate time it will take to complete them.

- Baseline eCRF (electronic Case Report Form) – 1.5 minutes
- Study specific questionnaire – 3 minutes
- AFEQT (AF Effect on Quality of Life) – 3 minutes
- JAKQ (Jessa Atrial Fibrillation Knowledge Questionnaire) – 3 minutes
- CALM (Confidence in Atrial fibrillation Management) – 2 minutes
- TBQ (Treatment Burden Questionnaire) – 3 minutes
- User experience survey: 1.5 minutes

5 Other relevant information about the research project

This project is being conducted at **[sites]** and we aim to involve 416 participants in this project.

This project is a follow-on study from the previous CHAT-AF pilot study, also conducted by WARC at Westmead Hospital.

6 Do I have to take part in this research project?

Participation in any research project is voluntary. If you do not wish to take part, you do not have to. If you decide to take part and later change your mind, you are free to withdraw from the project at any stage.

If you do decide to take part, you will be given this Participant Information and Consent Form to sign and you will be given a copy to keep. Your decision whether to take part or not to take part, or to take part and then withdraw, will not affect your routine treatment, your relationship with those treating you, or your relationship with **[site]**.

7 What are the alternatives to participation?

You do not have to take part in this research project to receive treatment at this hospital.

8 What are the possible benefits of taking part?

This research may not benefit you directly, but the potential benefits to the broader community outweigh the minimal risk of burdens. As part of this study, you may receive information regarding your AF, so that you can better manage your diagnosis outside of hospital. However, if you experience any distress due to the topics discussed, appropriate support will be arranged by the research team.

9 What are the possible risks and disadvantages of taking part?

While there is very low potential for any adverse events as a result of the study procedures, participants may feel distressed when discussing their symptoms and/or diagnosis of atrial fibrillation. Any health events arising will be reported as per ethics and governance protocols. If you become upset or distressed as a result of your participation in the research, appropriate support will be arranged by the research team.

10 What if new information arises during this research project?

Sometimes during the course of a research project, new information becomes available about the treatment that is being studied. If this happens, your study doctor will tell you about it and discuss with you whether you want to continue in the research project. If you decide to withdraw, your study doctor will make arrangements for your regular health care to continue. If you decide to continue in the research project you will be asked to sign an updated consent form.

Also, on receiving new information, your study doctor might consider it to be in your best interests to withdraw you from the research project. If this happens, he/ she will explain the reasons and arrange for your regular health care to continue.

11 Can I have other treatments during this research project?

This study does not involve changes to your treatment plan. You should tell the doctors involved in your care about any treatments you are receiving as part of your regular health care.

12 What if I withdraw from this research project?

If you do withdraw your consent during the research project, the study doctor and relevant study staff will not collect additional personal information from you, although personal information already collected will be retained to ensure that the results of the research project can be measured properly and to comply with law. You should be aware that data collected by the research team up to the time you withdraw will form part of the research project results. If you do not want them to do this, you must tell them before you join the research project.

Please call the Research Coordinator on [redacted] or email [redacted] if you wish to withdraw.

13 Could this research project be stopped unexpectedly?

We do not anticipate this project will stop before it is finished.

14 What happens when the research project ends?

The results of the project will be discussed and published within peer reviewed journals, at scientific meetings and in the media. The results will also be reported back to the ethics committee. In any publication or discussion, the information will be provided in such a way that you and other participants cannot be identified.

At the conclusion of the research project, a summary of results will be shared with participants as acknowledgement of their contributions to the study. No identifying information will be used in this summary.

Part 2 How is the research project being conducted?

15 What will happen to information about me?

By signing the consent form or providing verbal consent over the phone, you allow the study doctor and relevant research staff to collect and use personal information about you for the research project. Any information obtained in connection with this research project that can identify you will remain confidential.

Information about you may be obtained from your health records held at this and other health services for the purpose of this research. By signing the consent form, you agree to the research team accessing health records if they are relevant to your participation in this research project. Information about your participation in this research project may be recorded in your health records.

Your health information collected by your doctor and **[site]** is securely stored within **[site LHD]** computer network. This information will be accessed by the research team to conduct data analysis. Your information will only be used for the purpose of this research project and it will only be disclosed with your permission, except as required by the relevant law. All members of the research team are associated with the Western Sydney Local Health District.
When analysing data, any information that can identify you is removed and a study ID for example '001' is assigned to protect your identity. The type of information may include age, gender, ethnicity, your diagnosis, medications, and any other medical conditions and treatment plan.

We will also ask you for your contact information so we can contact you throughout the research project. For participants allocated to the "intervention" group contact information (first name, mobile number and preferred contact times) will be uploaded securely to Ericom-managed Genesys Cloud CX platform, a cloud hosted within Australia that we are using to deliver the digital support program. The Genesys platform initiates the phone call and hands off securely to Nuance Mix application that handles the call flow. The Nuance Mix application will be hosted on the Azure platform located in Australia region (two data centres – one in Sydney and one in Melbourne). The Australian Government's Australian Cyber Security Centre (ACSC) has awarded 'protected' certification to Azure cloud services, the highest data security certification available in Australia. All information about you will be stored in Australia.

It is expected that the results of this research project will be published and/or presented in a variety of forums. In any publication and/or presentation, information will be provided in such a

way that you cannot be identified. When we analyse the data, all personal information is excluded, and each participant is identified only by a random number. In this way no personal information is available when we conduct our analysis.

In accordance with relevant Australian and/or NSW privacy and other relevant laws, you have the right to request access to the information collected and stored by the research team about you. You also have the right to request that any information with which you disagree, be corrected. Please contact the research team member named at the end of this document if you would like to access your information.

Any information obtained for the purpose of this research project that can identify you will be treated as confidential and securely stored. It will be disclosed only with your permission, or as required by law.

Your health records and any information obtained, relevant to the study during the research project are subject to inspection (for the purpose of verifying the procedures and the data) by relevant study personnel or as required by law. By signing the Consent Form, you authorise release of, or access to, this confidential information to the relevant study personnel as noted above.

16 Complaints and compensation

If there is any complaint or concern regarding the conduct of this study, you should contact the complaints contact person listed at the end of this sheet.

Participants in this study will not receive payment but they will be making a contribution to improving care for others in similar situations.

17 Who is organising and funding the research?

The research project is being conducted by the WARC, a collaborative research centre established by the University of Sydney and the WSLHD. WARC is working in collaboration with the DHCRC, a government funded research centre with an aim to improve health engagement through the use of digital technology. The technology is provided by Nuance Communications. The project is a collaboration between Western Sydney Local Health District (of which Westmead Hospital is a part of), the University of Sydney, the Digital Health CRC and Nuance Communications.

No member of the research team will receive a personal financial benefit from your involvement in this research project (other than their ordinary wages).

18 Who has reviewed the research project?

All research in Australia involving humans is reviewed by an independent group of people called a Human Research Ethics Committee (HREC). The ethical aspects of this research project have been approved by the HREC of Western Sydney Local Health District.

This project will be carried out according to the *National Statement on Ethical Conduct in Human Research (2007)*. This statement has been developed to protect the interests of people who agree to participate in human research studies.

19 Further information and who to contact

The person you may need to contact will depend on the nature of your query.

If you want any further information concerning this project or if you have any medical problems which may be related to your involvement in the project (for example, any side effects), you can contact the principal study doctor, [redacted] on [redacted] or any of the following people:

Clinical contact person

Name	[insert PI]
Position	Principal Investigator
Telephone	[insert contact number]
Email	[insert contact number]

Clinical contact person (other)

Name	[insert name]
Position	Research Coordinator
Telephone	[redacted]
Email	[redacted]

For matters relating to research at the site at which you are participating, the details of the local site complaints person are:

Complaints contact person

Position	[insert site details]
Telephone	[insert site contact]
Email	[insert email address]

If you have any complaints about any aspect of the project, the way it is being conducted or any questions about being a research participant in general, then you may contact:

Reviewing HREC approving this research and HREC Executive Officer details

Reviewing HREC name	*Western Sydney Local Health District*
Executive Officer	[redacted]
Telephone	[redacted]
Email	[redacted]

Site RESEARCH GOVERNANCE Office contact

Position	[insert site details]
Telephone	[insert site contact]
Email	[insert email address]

Patient Representative for complaints

Position	[insert site details]
Telephone	[insert site contact]
Email	[insert email address]

Consent Form - *Adult providing own consent*

**Title**	Conversational HeAlth supporT in Atrial Fibrillation Self-Management
**Short Title**	CHAT-AF-S
**Coordinating Principal Investigator**	Professor Clara Chow
**Location**	**[site]**

Declaration by Participant

I have read the Participant Information Sheet or someone has read it to me in a language that I understand.

I understand the purposes, procedures and risks of the research described in the project.

I give permission for my doctors, other health professionals, hospitals or laboratories outside this hospital to release information to Westmead Applied Research Centre concerning my disease and treatment for the purposes of this project. I understand that such information will remain confidential.

I acknowledge that any regulatory authorities may have access to my medical records **specifically related** to this project to monitor the research in which I am agreeing to participate. However, I understand my identity will not be disclosed to anyone else or in publications or presentations.

I have had an opportunity to ask questions and I am satisfied with the answers I have received.

I freely agree to participate in this research project as described and understand that I am free to withdraw at any time during the study without affecting my future health care.

I understand that I will be given a signed copy of this document to keep.

Name of Participant (please print) ______________________________

Signature ______________________ Date ______________________

Name of Witness* to
Participant's Signature (please print) ______________________________

Signature ______________________ Date ______________________

* Witness is not to be the investigator, a member of the study team or their delegate. In the event that an interpreter is used, the interpreter may not act as a witness to the consent process. Witness must be 18 years or older.

Declaration by Study Doctor/Senior Researcher†

I have given a verbal explanation of the research project, its procedures and risks and I believe that the participant has understood that explanation.

Name of Study Doctor/
Senior Researcher† (please print) ______________________________

Signature ______________________ Date ______________________

† A senior member of the research team must provide the explanation of, and information concerning, the research project.

Note: All parties signing the consent section must date their own signature.

I understand that, if I decide to discontinue the study treatment, I may be asked to attend follow-up visits to allow collection of information regarding my health status. Alternatively, a member of the research team may request my permission to obtain access to my medical records for collection of follow-up information for the purposes of research and analysis.

Form for Withdrawal of Participation - *Adult providing own consent*

**Title**	Conversational HeAlth supporT in Atrial Fibrillation Self-Management
**Short Title**	CHAT-AF-S
**Coordinating Principal Investigator**	Professor Clara Chow
**Location**	**[site]**

Declaration by Participant

I wish to withdraw from participation in the above research project and understand that such withdrawal will not affect my routine treatment, my relationship with those treating me or my relationship with **[site]**.

Name of Participant (please print) ______________________________

Signature ______________________ Date ______________________

Declaration by Study Doctor/Senior Researcher†

I have given a verbal explanation of the implications of withdrawal from the research project and I believe that the participant has understood that explanation.

Name of Study Doctor/
Senior Researcher† (please print) ______________________________

Signature ______________________ Date ______________________

† A senior member of the research team must provide the explanation of and information concerning withdrawal from the research project.

Note: All parties signing the consent section must date their own signature.

Supplement 4. Information letter for General Practitioner

Address

Date:[xx/xx/xxx]

Dear [GP]

Your patient is enrolled in the CHAT-AF-S Clinical Trial

We would like to inform you that your patient has enrolled in the CHAT-AF-S (Conversational HeAlth support in Atrial Fibrillation Self-management) randomised controlled trial. The trial is evaluating the effectiveness of a 3-month digital health program comprising automated phone calls and text messages to improve self-management and quality of life among patients with atrial fibrillation (AF). Your patient was randomised to the intervention group.

This research study is being led by Professor Clara Chow, Cardiologist and Director of the University of Sydney's Westmead Applied Research Centre, and Associate Professor Liliana Laranjo. The study is approved by WSLHD Human Research Ethics Committee (HREC) ETH/00765.

Contact us

Please don't hesitate to contact the study team at warc.chat-af@sydney.edu.au or call us on +61 404 228 052 if you have any questions regarding this study.

Yours sincerely,

The CHAT-AF-S Study Team

Professor Clara Chow, Associate Professor Liliana Laranjo

Westmead Applied Research Centre, University of Sydney

Supplement 5. Central Monitoring Unit

The CHAT-AF-S Central Monitoring Unit operates following procedures detailed in the ‘CHAT-AF-S Manual of Procedures’ and ‘Health Counsellor Manual’. A team of Health Counsellors monitors participant responses and alerts. Health Counsellors are staff employed and trained by the Westmead Applied Research Centre (WARC) (having, at a minimum, an undergraduate Degree in a health-related/clinical field, e.g., nursing, allied health) and have contingent worker status at Westmead Hospital, in Western Sydney, where WARC is located.
The primary roles of the CHAT-AF-S Health Counsellors are to monitor and respond and/or escalate concerns, following procedures in the ‘CHAT-AF-S Manual of Procedures’ and ‘Health Counsellor Manual’. All alerts and potential issues are discussed by the Health Counsellor with the Study Director (a general practice doctor), as well as with the Chief Investigator (a cardiologist), as needed (at a minimum, at weekly team meetings).

Alerts are followed up with a phone call from the Health Counsellor within the next 2 business days, using predefined scripts (except in Table 1) and following procedures to attempt to reach participants if the initial phone call is unanswered. The Health Counsellor is also responsible for notifying the Study Director and/or Chief Investigator. The Study Director and/or Chief Investigator then decide on the appropriate action and escalation where needed, notifying the patient’s cardiologist.
All alerts and potential issues are documented, along with details regarding communication attempts (mode of contact, date, time), and the content of conversations with participants, including any advised actions and responses.

Table 1: Adapted excerpts from call scripts with triggered alerts and health counsellor responses

**Outreach**	**Call script excerpt and responses triggering alerts**	**Health Counsellor Response (1-2 business days)**
**1**	“…On a scale of 1 to 5, where 1 is very poor and 5 is excellent, how would you rate your health?”<br><br>Participant response: 1, 2 (Very poor, Poor)<br><br>“We are sorry to hear that, would you like to talk to someone in our care team?”<br><br>Participant response: Yes<br><br>“…Okay we'll call you in the next couple of days. Of course, if you feel very unwell, please seek medical attention immediately.”	Hi <xxx>, this is <xx> calling from the CHAT-AF-S study / X Hospital Atrial Fibrillation program. How are you?<br>We are following up because in the last call you responded your overall health was poor (1 or 2)?<br>Prompt 1: What are you experiencing with your health at the moment?<br>Prompt 2: Are you in touch with your GP or Cardiologist about your health?<br>Advise to see their GP/doctor or to seek medical attention immediately in case they feel very unwell.
	“…Now we want to talk to you about medications, which are an important part of managing your Atrial Fibrillation. We'd like to check, are you taking blood thinning medicines for your Atrial Fibrillation? You can say ‘Yes’, ‘No’ or ‘Not sure’.”	II) Hi <xxx>, this is <xx> calling from the CHAT-AF-S study / X Hospital Atrial Fibrillation program. How are you?<br>We’re calling you because you indicated you had questions about whether you are on a blood thinner medication?<br>Prompt 1: Do you have a medication list or a blister pack? What medications are you on?

	Participant response: Not sure<br><br>“Okay, it might be good to raise this with your GP or doctor at your next visit. Would you like to speak to someone from our team about your medications?”<br><br>Participant response: Yes<br><br>“We will call you in the next few days. Look out for our text messages and videos from our heart specialists with more information about Atrial Fibrillation medicines.”	Prompt 2: I’m going to read out a few names, let me know if you can find them on the list? List: Warfarin, apixaban (Eliquis), dabigatran (Pradaxa) and rivaroxaban (Xarelto)...<br>Prompt 3: Blood thinner medication or anticoagulants are important to prevent a stroke. You can talk to your GP or Cardiologist about these medications and ask them any questions you may have.
**2**	“... If you are experiencing symptoms, would you like to talk to someone from our team?”<br><br>Participant response: Yes<br><br>“Okay we will call you in the next couple of days. Of course, if you feel very unwell, please seek medical attention immediately.”	Hi <xxx>, this is <xx> calling from the CHAT-AF-S study / X Hospital Atrial Fibrillation program. We are checking in because you mentioned you wanted to talk to someone about the symptoms you are experiencing.<br>Prompt 1: What symptoms are you experiencing? Start date, frequency, duration, intensity...<br>Prompt 2: Have you discussed these symptoms with your GP or Cardiologist? They can advise on medications or procedures to help with the symptoms.<br>Prompt 3: Advise patient to see their GP or to seek medical attention immediately in case they feel very unwell.
**4**	“...do you feel like you can manage your Atrial Fibrillation symptoms? Please say ‘yes’, ‘no’ or ‘not sure’.”<br><br>Participant response: No/Not sure<br><br>“If you’re not feeling on top of your symptoms, or you’re not sure how to manage your triggers, talk to your doctor and include some options in your Atrial Fibrillation action plan. Would you like to talk to someone from our team?”<br><br>Participant response: Yes<br><br>“Okay, someone will call you in a couple of days. Of course, if you feel very unwell, please seek medical attention immediately.”<br><br>Participant response: No<br><br>“Okay, we suggest you see your General Practitioner.”	Hi <xxx>, this is <xx> calling from the CHAT-AF-S study / X Hospital Atrial Fibrillation program.<br>We are checking in because in the last call, you responded you were struggling to manage your atrial fibrillation symptoms?<br>Prompt 1: What symptoms are you experiencing? Start date, frequency, duration, intensity...<br>Prompt 2: Have you managed to talk to your GP or cardiologist about it? Do you have an action plan?<br>Prompt 3: Advise them to see their GP or Cardiologist, or to seek medical attention immediately in case they feel very unwell.

Supplement 6. CHAT-AF-S behaviour change features

**COM-B model component**[3, 4]		**CHAT-AF-S features and BCTs**
**Capability**	Psychological capability	Micro-learning through bite-sized education from credible source; information about health consequences and outcomes; action plan template; symptoms and triggers monitoring template; empowerment and shared decision-making support (eg "what to ask/discuss with doctor")
	Physical capability	Instruction on how to perform behaviours (eg measuring blood pressure)
**Opportunity**	Physical opportunity	Access to clinician escalation where needed, via call alerts and clinical call back
	Social opportunity	Clinician reinforcement and support via call back
**Motivation**	Reflective motivation	Goal setting and action planning support; prompting discussions with doctors about preferences and goals
	Automatic motivation	Suggestions for pairing behaviours (eg taking medication) with daily routines to form self-management habits; support for dealing with the emotional impact of AF

Abbreviations: AF, Atrial Fibrillation; BCT, behaviour change technique; COM-B, Capability, Opportunity, Motivation, Behaviour model

[3] Michie S, Richardson M, Johnston M, Abraham C, Francis J, Hardeman W, et al. The behavior change technique taxonomy (v1) of 93 hierarchically clustered techniques: building an international consensus for the reporting of behavior change interventions. Annals of behavioral medicine 2013;46(1):81-95
[4] Michie S, Van Stralen MM, West R. The behaviour change wheel: a new method for characterising and designing behaviour change interventions. Implementation science 2011;6(1):42

Supplement 7. Study-specific questionnaire

**Smoking**	
Current smoker (have you smoked in last month?)	Yes<br>No
If yes, tick one or more	Cigarettes<br>Electronic cigarettes<br>Other
**Alcohol**	
Do you drink alcohol?	Yes<br>No
If yes, how many standard drinks do you typically have per week?<br><br>Select one<br>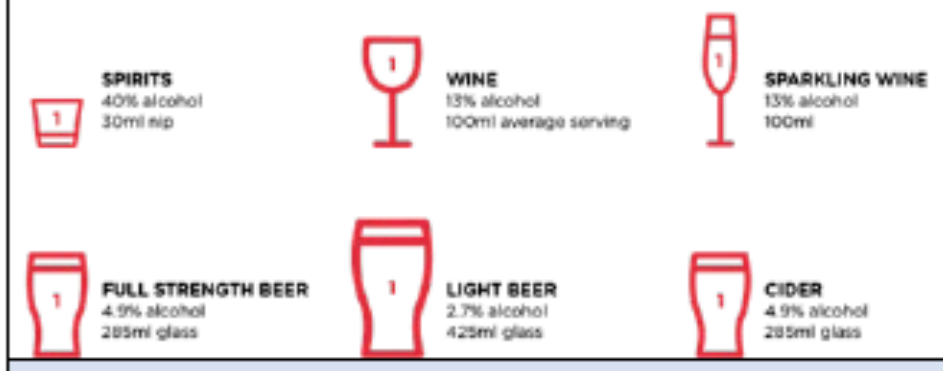	1 or less/week<br>2-4/week<br>5-9 /week<br>10 or more/week
**Physical Activity**	
In the last 7 days, in total how many hours/minutes did you spend being physically active for sport, exercise or recreation?	__ Hours __ Minutes
In the last 7 days, in total how many hours/minutes did you spend being physically active for work (in a way that raises your heart rate and makes you breathe faster, so that you might still be able to talk but not comfortably sing if you wanted to)?	__ Hours __ Minutes
**Diet**	
Over the LAST ONE MONTH ONLY, on average, how many servings of **vegetables/legumes** did you eat per day?<br>**What is a serve of vegetables?**<br>**A standard serve is about 75g (100–350kJ) or:**<br>½ cup cooked green or orange vegetables (for example, broccoli, spinach, carrots or pumpkin)<br>½ cup cooked dried or canned beans, peas or lentils (preferably with no added salt)<br>1 cup green leafy or raw salad vegetables<br>½ cup sweet corn<br>½ medium potato or other starchy vegetables (sweet potato, taro or cassava)<br>1 medium tomato	None<br>Less than 1 serving per day<br>1 serving per day<br>2 servings per day<br>3 servings per day<br>4 servings per day<br>5 servings per day<br>6 servings per day<br>7 or more servings per day
Over the LAST ONE MONTH ONLY, on average, how many servings of **fruit** did you eat per day?<br>**What is a serve of fruit?**<br>**A standard serve is about 150g (350kJ) or:**<br>1 medium apple, banana, orange or pear<br>2 small apricots, kiwi fruits or plums<br>1 cup diced or canned fruit (no added sugar)	None<br>Less than 1 serving per day<br>1 serving per day<br>2 servings per day<br>3 servings per day<br>4 or more servings per day
**Weight**	
What is your current weight (kg)	
**Healthcare service utilisation**	
In the last 2/6 months, how many times have you used the following services.	

(Please include telehealth appointments).<br>If not seen, enter 0.	
General Practice (GP)	
Cardiologist Specialist (Heart Doctor)	
Emergency Department	
Hospital stays due to cardiovascular reason<br>(heart or blood vessels problem)	
If yes, please indicate which of the below apply:	a) heart attack,<br>b) stroke,<br>c) heart rhythm problem<br>d) cardiac surgery or cardiac procedure (please specify)<br>e) other (please specify)
**Medication adherence**	
Specific to your atrial fibrillation medication, in the past month, how often did you take your medications as the doctor prescribed?	All of the time (100%)<br>Nearly all of the time (90%)<br>Most of the time (75%)<br>About half the time (50%)<br>Less than half the time (<50%)<br>No atrial fibrillation medication
Specific to your atrial fibrillation medication, in the past month, how often did you <u>forget</u> to take one or more of your prescribed medications?	Never<br>Once<br>2-3 times<br>Once per week<br>Several times per week<br>Nearly every day<br>No atrial fibrillation medication
Specific to your atrial fibrillation medication, in the past month, how often did you decide to <u>skip</u> one or more of your prescribed medications?	Never<br>Once<br>2-3 times<br>Once per week<br>Several times per week<br>Nearly every day<br>No atrial fibrillation medication

References

1. Rivera SC, Liu X, Chan A-W, Denniston AK, Calvert MJ, Ashrafian H, et al. Guidelines for clinical trial protocols for interventions involving artificial intelligence: the SPIRIT-AI extension. The Lancet Digital Health 2020;2(10):e549-e560.
2. Agarwal S, LeFevre AE, Lee J, L'engle K, Mehl G, Sinha C, et al. Guidelines for reporting of health interventions using mobile phones: mobile health (mHealth) evidence reporting and assessment (mERA) checklist. bmj 2016;352.
3. Michie S, Richardson M, Johnston M, Abraham C, Francis J, Hardeman W, et al. The behavior change technique taxonomy (v1) of 93 hierarchically clustered techniques: building an international consensus for the reporting of behavior change interventions. Annals of behavioral medicine 2013;46(1):81-95.
4. Michie S, Van Stralen MM, West R. The behaviour change wheel: a new method for characterising and designing behaviour change interventions. Implementation science 2011;6(1):42.
