# Supplementary material for: Conversational artificial intelligence HeAlth supporT in Atrial Fibrillation Self-Management (CHAT-AF-S): rationale and randomised controlled trial design
Source: BMJ Open. 2026 Aug 7;16(8):e120834. doi: 10.1136/bmjopen-2026-120834 (PMC13475244; doi:10.1136/bmjopen-2026-120834)
Supplement: Supplementary file 2 [file bmjopen-2026-120834.reviewer_comments.pdf]

PEER REVIEW HISTORY

BMJ Open publishes all reviews undertaken for accepted manuscripts. Reviewers are asked to complete a checklist review form and are provided with free text boxes to elaborate on their assessment. These free text comments are reproduced below.

ARTICLE DETAILS

Title (Provisional)

Conversational artificial intelligence HeAlth supporT in Atrial Fibrillation Self-Management (CHAT-AF-S): rationale and randomised controlled trial design

Authors

Laranjo, Liliana; Zeng, Aileen; O'Hagan, Edel; Trivedi, Ritu; Sathiaraj, Rahul; Thomas, Stuart; Thiagalingam, Aravinda; Kovoor, Pramesh; Sivagangabalan, Gopal; Kizana, Eddy; Kumar, Saurabh; Kilian, Jens; Marschner, Simone; Shaw, Tim J; Chow, Clara

---

VERSION 1 - REVIEW

**Reviewer**	**1**
**Name**	**Darroudi, Susan**
**Affiliation**	**Mashhad University of Medical Sciences**
**Date**	**05-May-2026**
**COI**	**None**

The paper entitled "Conversational artificial intelligence HeAlth supporT in Atrial Fibrillation Self-Management (CHAT-AF-S): rationale and randomised controlled trial design" presents a well-designed and methodologically robust study addressing an important clinical question: the effectiveness of a conversational AI–based intervention in supporting self-management among patients with atrial fibrillation. The study builds appropriately on prior pilot work and adheres to established reporting standards (SPIRIT-AI, mERA). The co-designed, theory-informed intervention—grounded in the Behaviour Change Wheel and AF-CARE framework—and its integration into clinical care through clinician escalation pathways represent notable strengths. With recruitment nearly complete (n=481 as of December 2025), the manuscript would benefit primarily from clarifications and enhancements in reporting rather than substantive design modifications.

However, there are several issues need to addressed

1. The manuscript states recruitment ended in December 2025 and "Database lock and analysis are planned for early 2026." Given the submission date (April 2026) and current

date, the authors should clarify whether database lock has occurred and whether any preliminary results are known.

2. The 3-month primary endpoint is relatively short for a quality-of-life intervention, especially given that two prior larger trials of a similar (non-AI) relational agent failed to show benefit at 12 months despite a positive 1-month signal in a smaller trial. The authors should explicitly justify why 3 months is the appropriate timepoint and acknowledge that durability of effect cannot be assessed. Are there plans for longer-term follow-up (6 or 12 months) even if not powered as the primary outcome?

3. The power calculation assumes a between-group difference of 7 points on AFEQT (SD=26), while a 5-point difference is described as the minimum clinically important difference. Powering for a 7-point effect rather than the MCID risks missing clinically meaningful but smaller effects. Please justify the choice of 7 points as the target effect size and discuss whether the trial would be considered "negative" if the observed difference falls between 5 and 7 points.

4. The protocol mentions a "central monitoring unit" and clinical escalation following alerts, but details are sparse. Please clarify:

• Who staffs the central monitoring unit, their qualifications, hours of operation, and turnaround time guarantees beyond the stated "two days."

• The decision algorithm for escalation (criteria for callback vs. urgent referral vs. emergency services).

• How acute symptoms reported during a call (chest pain, syncope, stroke symptoms) are handled in real time, given that automated calls may be answered when the patient is alone.

• Whether there is any out-of-hours coverage, and what participants are instructed to do for urgent issues.

5. Patients are recruited from two hospitals where some treating cardiologists may see both arms. There is no discussion of how contamination (clinicians changing usual care behavior based on the trial) will be assessed or minimized. Please address this.

6. Eligibility criterion 4 ("able to receive text messages and open weblinks embedded in them") should specify how this is operationalized — self-report, demonstration, or smartphone ownership? This affects generalisability.

7. Recruitment via inpatients ready for discharge may capture patients during a vulnerable period when QoL baseline may be artificially low (post-acute event), inflating apparent improvement. Consider stratifying or reporting baseline QoL by recruitment setting (inpatient vs. outpatient).

8. Engagement metric (≥4 of 6 outreaches = 70% adherence threshold). 4/6 = 66.7%, not 70%. Please correct the arithmetic or threshold.

9. The protocol cites both the MRC framework and NASSS framework but does not clearly map which questions each framework addresses. A brief sentence on how they are integrated would help.

10. "Minimum of 20 patients" with interviews continuing until "conceptual depth" is reached is appropriate, but the authors cite both saturation-based references (Guest 2006) and saturation-critical references (Braun & Clarke 2021). A brief statement on the chosen rationale (information power, conceptual depth) would clarify the position.

11. The exploratory complier-average causal effect analysis is welcome but needs more detail: how will "compliance" be operationalized (binary at 70% threshold? continuous?), and what are the assumptions (exclusion restriction, monotonicity)?

12. Healthcare utilization is a secondary outcome but the data source is not specified (self-report vs. record linkage). Self-reported utilization over 3 months has known recall bias.

13. The protocol mentions stratification by sex and site but the analysis section says stratification variables will be included as fixed effects in dichotomous outcome models please confirm the same applies for the ANCOVA models for continuous outcomes.

14. The abstract methods state "We will randomize 480 adults" while the ethics/dissemination section reports 481 already recruited. Please reconcile (briefly note the slight over-recruitment in the dissemination paragraph).

15. The figures referenced in the manuscript (Figures 1–3) appear at low resolution in the proof and contain dense information; ensure final versions are legible at print size, especially Figure 3 (the table of weekly content).

16. Reference 54 is a placeholder ("Conversational artificial intelligence...In; 2026") — please provide the OSF URL/DOI.

17. Some references use full journal names while others use abbreviations; please standardise per BMJ Open style.

18. Line 273: "Medical Research Council framewor51" — typo (missing "k").

19. Line 64–65: "but randomized controlled trials remain sparse" — clarify "remain sparse for conversational AI in chronic disease self-management" for precision.

20. Throughout: inconsistent capitalization of "CHAT-AF-S" intervention components (e.g., "Conversational HeAlth supporT" vs. "Conversational Health support" in Supplement 3).

The manuscript could be further strengthened by situating the intervention within the broader landscape of emerging AI-enabled health systems and conversational technologies, for example by citing recent work on privacy-preserving mobile health platforms, conversational modeling, and AF-related mechanistic insights (Non-Interactive Privacy-Preserving System for Heterogeneous Mobile Health Diagnosis, GTS-21 alleviates sepsis-induced atrial fibrillation susceptibility by modulating macrophage polarization and

Neuregulin-1 secretion, multi-message passing framework based on heterogeneous graphs in conversational emotion recognition)

Reviewer **2**
Name **Magalhaes, Teresa**
Affiliation **NOVA University Lisbon, NOVA National School of Public Health**
Date **13-May-2026**
COI **None**

Dear Authors,

I would like to congratulate you on your study and on the importance of this topic in generating clinical evidence and demonstrating clear benefits for patients in the field of digital health. This randomised clinical trial is very well written and planned. I have nothing to add, other than to note that the timing of the protocol submission seems late to me, given the start date for recruitment and the date of the first results in early 2026. I found just one typo on line 273, page 14, in “framewor".

Reviewer **3**
Name **Gallagher, Celine**
Affiliation **Adelaide University, Australian Dysautonomia and Arrhythmia Research Collaborative**
Date **09-Jun-2026**
COI **None**

The manuscript entitled 'Conversational artificial intelligence HeAlth supporT in Atrial Fibrillation Self-Management (CHAT-AF-S): rationale and randomised controlled trial design' was submitted for review. Overall, this is a well designed study that will be an important contribution to current literature in this area.

Major issues:

- Please confirm that this study is still ongoing, as it would not be appropriate to publish a protocol on a study that has been completed.

- Please comment on the use of AFEQT as a primary outcome measure. How was this determined? Was there any lived experience engagement in the choice or primary outcome measure?

- Are you able to provide further details as to how patient engagement was embedded in to your trial e.g. was a consumer group recruited, how many were recruited, was this through workshops etc)

- Was there any limitations placed on AF type for inclusion in the study (paroxysmal vs persistent vs permanent)? If so, was any randomisation stratification undertaken for this?

- Was inclusion in a current cardiac rehabilitation program a reason for exclusion from the trial?

- Are you able to provide any commentary on standard of care at the recruiting institutions with respect to education and risk factor management?

Minor issues:

- I would suggest a longer duration for qualitative interviews e.g. 30-60 minutes per participant.

VERSION 1 - AUTHOR RESPONSE

**Reviewer 1**	
The paper entitled "Conversational artificial intelligence HeAlth supporT in Atrial Fibrillation Self-Management (CHAT-AF-S): rationale and randomised controlled trial design" presents a well-designed and methodologically robust study addressing an important clinical question: the effectiveness of a conversational AI–based intervention in supporting self-management among patients with atrial fibrillation. The study builds appropriately on prior pilot work and adheres to established reporting standards (SPIRIT-AI, mERA). The co-designed, theory-informed intervention—grounded in the Behaviour Change Wheel and AF-CARE framework—and its integration into clinical care through clinician escalation pathways represent notable strengths. With recruitment nearly complete (n=481 as of December 2025), the manuscript would benefit primarily from clarifications and enhancements in reporting rather than substantive design modifications. However, there are several issues need to addressed	Thank you for reviewing our submission and for your positive assessment of the study design and methodology. We have addressed the specific issues raised in the comments below.
1. The manuscript states recruitment ended in December 2025 and "Database lock and analysis are planned for early 2026." Given the submission date (April 2026) and current date, the authors should clarify whether	Delays in finalising data collection and patient follow-up procedures lasting up to May 2026 have delayed database lock and analysis beyond what was initially expected. We have modified the text accordingly to reflect that database lock and analysis are now planned for mid 2026.

database lock has occurred and whether any preliminary results are known.	Modified as suggested in the manuscript:<br>"Database lock and analysis are planned for mid 2026."
2. The 3-month primary endpoint is relatively short for a quality-of-life intervention, especially given that two prior larger trials of a similar (non-AI) relational agent failed to show benefit at 12 months despite a positive 1-month signal in a smaller trial. The authors should explicitly justify why 3 months is the appropriate timepoint and acknowledge that durability of effect cannot be assessed. Are there plans for longer-term follow-up (6 or 12 months) even if not powered as the primary outcome?	Thank you for this comment. Three months was chosen as the timepoint to assess the initial impact of the 3-month CHAT-AF-S intervention, based on our feasibility constraints and project budget. Similar timepoints have been used for AFEQT assessment as primary outcome in clinical trials:<br>- Marcus G et al. Individualized studies of triggers of paroxysmal atrial fibrillation: the I-STOP-AFib randomized clinical trial. JAMA cardiology. 2022 Feb;7(2):167-74. *The primary outcome comparing the n-of-1 intervention and control groups was the AFEQT score at* ***10 weeks****.*<br>- Särnholm J, et al. Cognitive behavioral therapy improves quality of life in patients with symptomatic paroxysmal atrial fibrillation. Journal of the American College of Cardiology. 2023 Jul 4;82(1):46-56. *The primary outcome was the AFEQT summary score at* ***3-month*** *follow-up.*<br><br>We have added the following to the methods section to clarify this decision:<br>"Choice of the primary outcome follows international guideline recommendations for assessment of quality-of-life and patient-reported outcomes in AF clinical trials[4]; this choice was further guided by our previous pilot trial 'Coordinating Healthcare with Artificial intelligence Technology for patients with Atrial Fibrillation (CHAT-AF)', where we found a significant pre-post improvement in AFEQT overall score in the intervention group[30, 31], and was informed by lived experience (qualitative interviews with pilot participants and co-author investigator living with AF)[32]. The 3-month endpoint reflects the pragmatic decision to focus on short-term impact and measure the initial effect of the 3-month CHAT-AF-S intervention on the AFEQT overall score, within feasibility constraints."
3. The power calculation assumes a between-group difference of 7 points on AFEQT (SD=26), while a 5-point difference is described as the minimum clinically important difference. Powering for a 7-point effect rather than the MCID risks missing clinically meaningful but smaller effects.	We acknowledge that an observed difference between 5 and 7 points would still be clinically meaningful, and this will be considered in the interpretation of the trial results. We have clarified our rationale for powering the study for a 7-point difference, as highlighted below:

Please justify the choice of 7 points as the target effect size and discuss whether the trial would be considered "negative" if the observed difference falls between 5 and 7 points.	"With a difference of 5 being considered the minimal clinically important difference in AFEQT overall score[27-29], and based on feasibility constraints and results from previous trials[50, 51], including our previous pilot study, a difference of 7 was used for the sample size calculation."
4. The protocol mentions a "central monitoring unit" and clinical escalation following alerts, but details are sparse. Please clarify:<br>• Who staffs the central monitoring unit, their qualifications, hours of operation, and turnaround time guarantees beyond the stated "two days."<br>• The decision algorithm for escalation (criteria for callback vs. urgent referral vs. emergency services).<br>• How acute symptoms reported during a call (chest pain, syncope, stroke symptoms) are handled in real time, given that automated calls may be answered when the patient is alone.<br>• Whether there is any out-of-hours coverage, and what participants are instructed to do for urgent issues.	We have provided additional details regarding the central monitoring unit, including the call back process from health counsellors and clinical escalation as needed, in Supplement 5:<br><br>Excerpts from Supplement 5: Central Monitoring Unit<br>The CHAT-AF-S Central Monitoring Unit operates following procedures detailed in the 'CHAT-AF-S Manual of Procedures' and 'Health Counsellor Manual'. A team of Health Counsellors monitors participant responses and alerts. Health Counsellors are staff employed and trained by the Westmead Applied Research Centre (WARC) (having, at a minimum, an undergraduate Degree in a health-related/clinical field, e.g., nursing, allied health) and have contingent worker status at Westmead Hospital, in Western Sydney, where WARC is located.<br>The primary roles of the CHAT-AF-S Health Counsellors are to monitor and respond and/or escalate concerns, following procedures in the 'CHAT-AF-S Manual of Procedures' and 'Health Counsellor Manual'. All alerts and potential issues are discussed by the Health Counsellor with the Study Director (a general practice doctor), as well as with the Chief Investigator (a cardiologist), as needed (at a minimum, at weekly team meetings). Alerts are followed up with a phone call from the Health Counsellor within the next 2 business days, using predefined scripts (except in Table 1) and following procedures to attempt to reach participants if the initial phone call is unanswered. The Health Counsellor is also responsible for notifying the Study Director and/or Chief Investigator. The Study Director and/or Chief Investigator then decide on the appropriate action and escalation where needed, notifying the patient's cardiologist.<br>All alerts and potential issues are documented, along with details regarding communication attempts (mode of contact, date, time), and the content of conversations with participants, including any advised actions and responses.<br><br>Table 1: Adapted excerpts from call scripts with triggered alerts and health counsellor responses

	[Example from call 4: "…do you feel like you can manage your Atrial Fibrillation symptoms? Please say 'yes', 'no' or 'not sure'."<br><br>Participant response: No/Not sure<br><br>"If you're not feeling on top of your symptoms, or you're not sure how to manage your triggers, talk to your doctor and include some options in your Atrial Fibrillation action plan. Would you like to talk to someone from our team?"<br><br>Participant response: Yes<br><br>"Okay, someone will call you in a couple of days. Of course, if you feel very unwell, please seek medical attention immediately."<br><br>--<br>Health Counsellor call back example: Hi <xxx>, this is <xx> calling from the CHAT-AF-S study / X Hospital Atrial Fibrillation program.<br>We are checking in because in the last call, you responded you were struggling to manage your atrial fibrillation symptoms?<br>Prompt 1: What symptoms are you experiencing? Start date, frequency, duration, intensity…<br>Prompt 2: Have you managed to talk to your GP or cardiologist about it? Do you have an action plan?<br>Prompt 3: Advise them to see their GP or Cardiologist, or to seek medical attention immediately in case they feel very unwell.<br><br>[see table 1 for additional details]
5. Patients are recruited from two hospitals where some treating cardiologists may see both arms. There is no discussion of how contamination (clinicians changing usual care behavior based on the trial) will be assessed or minimized. Please address this.	We acknowledge the possibility of contamination and differential clinician behaviour and have added a paragraph on this, as suggested:<br>"No information on intervention content will be provided to cardiologists in recruiting sites and only participants allocated to the intervention group will be able to access the intervention, as phone calls and text messages are delivered directly to their personal phone number. Although unlikely, contamination will be assessed using intervention access records and logs of communications with participants and clinicians; any contamination will be summarised overall, by intervention component and site. An exploratory Complier Average Treatment Effect (CATE) analysis (described below) will also reflect receipt of intervention in each group. All analyses will be adjusted for site of recruitment and sex (stratification variables)."
6. Eligibility criterion 4 ("able to receive text messages and open weblinks embedded in	We have clarified that this criterion is operationalized through self-report:

them") should specify how this is operationalized — self-report, demonstration, or smartphone ownership? This affects generalisability.	"4) are able to receive text messages and open weblinks embedded in them (self-reported)"
7. Recruitment via inpatients ready for discharge may capture patients during a vulnerable period when QoL baseline may be artificially low (post-acute event), inflating apparent improvement. Consider stratifying or reporting baseline QoL by recruitment setting (inpatient vs. outpatient).	We agree with this point and have added the following to the data analysis section in the manuscript: "We will explore heterogeneity of treatment effect by sociodemographic characteristics, CHA2DS2VA score, recency of AF diagnosis, type of AF, and recruitment setting (inpatient versus outpatient); results will be presented displaying stratification of the outcome by subgroup."
8. Engagement metric (≥4 of 6 outreaches = 70% adherence threshold). 4/6 = 66.7%, not 70%. Please correct the arithmetic or threshold.	Thank you for pointing this out. We have corrected it in the manuscript: "Adherence will be defined as meeting the minimum intervention use threshold of 4 or more outreaches completed out of 6"
9. The protocol cites both the MRC framework and NASSS framework but does not clearly map which questions each framework addresses. A brief sentence on how they are integrated would help.	We have clarified how the MRC framework and NASSS framework will be used to guide the qualitative interviews and process evaluation: "The MRC framework[55] will guide exploration of how CHAT-AF-S was implemented and experienced, including engagement, acceptability (e.g., satisfaction with aspects of the intervention such as content, complexity, and delivery), usability, appropriateness (e.g., relevance, usefulness, practicability), perceived utility, and contextual barriers and enablers. The Non-adoption, Abandonment, Scale-up, Spread and Sustainability (NASSS) framework[57] will guide questions examining factors influencing non-adoption or abandonment and the intervention's potential for integration into routine care, scale-up, spread, and longer-term sustainability."
10. "Minimum of 20 patients" with interviews continuing until "conceptual depth" is reached is appropriate, but the authors cite both saturation-based references (Guest 2006) and saturation-critical references (Braun & Clarke 2021). A brief statement on the chosen rationale (information power, conceptual depth) would clarify the position.	We have clarified our chosen rationale, aligning with the concept of "conceptual depth" for our thematic analysis approach; we have removed the Guest 2006 reference.
11. The exploratory complier-average causal effect analysis is welcome but needs more detail: how will "compliance" be operationalized (binary at 70% threshold? continuous?), and what are the assumptions (exclusion restriction, monotonicity)?	We have provided more details on the Complier Average Treatment Effect (CATE) analysis, including the operationalization of compliance and the underlying assumptions: "An exploratory secondary analysis will estimate the Complier Average Treatment Effect (CATE)[54, 55] to quantify the causal effect of adhering to the CHAT-AF-S intervention on the primary outcome, overall AFEQT score, provided key assumptions

	are met (e.g., exclusion restriction; monotonicity; Stable Unit Treatment Value Assumption). Adherence ("treatment compliance") will be defined a priori as meeting the minimum intervention use threshold of 4 or more outreaches completed out of 6. We will implement a two-stage least squares analysis: in the first stage, adherence (binary) will be modelled as a function of randomised assignment, and in the second stage, overall AFEQT at follow-up will be regressed on the predicted adherence from stage 1. We will also complete sensitivity analyses, defining adherence as completing 5 out of 6 outreaches and 3 out of 6 of the outreaches, as well as using a continuous adherence measure that combines outreach completion with text message engagement."<br><br>Assumptions will be assessed prior to analysis. The monotonicity assumption is most likely met as only participants allocated to the intervention group will be able to access the intervention, as added below:<br>"only participants allocated to the intervention group will be able to access the intervention, as phone calls and text messages are delivered directly to their personal phone number. Although unlikely, contamination will be assessed using intervention access records and logs of communications with participants and clinicians; any contamination will be summarised overall, by intervention component and site."<br><br>The exclusion restriction assumption will need careful assessment, particularly in the case of the binary outreach-completion adherence threshold. Therefore, we added a sensitivity analysis with a continuous adherence measure that will combine outreach completion with text message engagement, as below:<br>"We will also complete sensitivity analyses, defining adherence as completing 5 out of 6 outreaches and 3 out of 6 of the outreaches, as well as using a continuous adherence measure that combines outreach completion with text message engagement."
12. Healthcare utilization is a secondary outcome but the data source is not specified (self-report vs. record linkage). Self-reported utilization over 3 months has known recall bias.	We have now specified this in the manuscript:<br><br>"self-reported healthcare utilisation"
13. The protocol mentions stratification by sex and site but the analysis section says stratification variables will be included as fixed effects in dichotomous outcome models	Thank you for pointing out this oversight, which has now been corrected as below:<br><br>"For the primary and secondary continuous outcomes, groups will be compared at 3 months

please confirm the same applies for the ANCOVA models for continuous outcomes.	using Analysis of Covariance (ANCOVA), adjusted for corresponding baseline values and stratification variables."
14. The abstract methods state "We will randomize 480 adults" while the ethics/dissemination section reports 481 already recruited. Please reconcile (briefly note the slight over-recruitment in the dissemination paragraph).	We have added a brief note acknowledging this, as suggested:<br>"Recruitment started in November 2023, and as of December 2025 a total of 481 patients were recruited (slight over-recruitment above the planned sample size of 480)."
15. The figures referenced in the manuscript (Figures 1–3) appear at low resolution in the proof and contain dense information; ensure final versions are legible at print size, especially Figure 3 (the table of weekly content).	We have provided high-resolution versions of Figures 1-3 to ensure they are fully legible at print size.
16. Reference 54 is a placeholder ("Conversational artificial intelligence...In; 2026") — please provide the OSF URL/DOI.	We have updated this reference (now reference 20):<br>"20.Conversational artificial intelligence HeAlth supporT in Atrial Fibrillation Self-Management (CHAT-AF-S). In Open Science Framework https://osf.io/phk54/overview?view_only=3d84fd823a9c4b908fad980cf565df36; 2026."
17. Some references use full journal names while others use abbreviations; please standardise per BMJ Open style.	We have standardized all references according to the BMJ Open style.
18. Line 273: "Medical Research Council framewor51" — typo (missing "k").	We have corrected this typo.
19. Line 64–65: "but randomized controlled trials remain sparse" — clarify "remain sparse for conversational AI in chronic disease self-management" for precision.	We have revised the text as suggested:<br>"remain sparse for conversational AI in chronic disease self-management"
20. Throughout: inconsistent capitalization of "CHAT-AF-S" intervention components (e.g., "Conversational HeAlth supporT" vs. "Conversational Health support" in Supplement 3).	We have carefully reviewed and ensured consistent capitalization of the "CHAT-AF-S" acronym throughout the manuscript and supplements.
The manuscript could be further strengthened by situating the intervention within the broader landscape of emerging AI-enabled health systems and conversational technologies, for example by citing recent work on privacy-preserving mobile health platforms, conversational modeling, and AF-related mechanistic insights (Non-Interactive Privacy-Preserving System for Heterogeneous Mobile Health Diagnosis, GTS-21 alleviates sepsis-induced atrial fibrillation susceptibility by modulating macrophage polarization and Neuregulin-1 secretion, multi-message passing framework based on heterogeneous graphs in conversational emotion recognition)	We have updated the introduction to add relevant recently published literature, as below.<br><br>"Isakadze N, Armoundas A, Sanders P, Andrade JG, Baykaner T, Lorvidhaya P, et al. Digital health tools for patient-centered lifestyle and risk factor modification in atrial fibrillation management. Heart Rhythm 2026."<br><br>We thank the reviewer for the suggested references. After careful consideration, we have not included them because they address highly specialised technical topics that fall outside the scope and primary focus of the present manuscript. We believe that incorporating these references would require additional technical discussion that is not necessary to support our study's aims or findings and may detract from the manuscript's central message. We have therefore retained references

	that are directly relevant to the research question and the level of technical detail appropriate for the intended readership.
**Reviewer 2**	
I would like to congratulate you on your study and on the importance of this topic in generating clinical evidence and demonstrating clear benefits for patients in the field of digital health. This randomised clinical trial is very well written and planned. I have nothing to add, other than to note that the timing of the protocol submission seems late to me, given the start date for recruitment and the date of the first results in early 2026. I found just one typo on line 273, page 14, in "framewor".	Thank you for your positive feedback and support for our study. We acknowledge the timing of the protocol submission; it was submitted as soon as feasible while ensuring all methodological details were thoroughly finalized.<br>Additional delays in finalising data collection and patient follow-up procedures lasting up to May 2026 have delayed database lock and analysis beyond what was initially expected. We have modified the text accordingly to reflect that database lock and analysis are now planned for mid 2026:<br>"Database lock and analysis are planned for mid 2026."
**Reviewer 3**	
The manuscript entitled 'Conversational artificial intelligence HeAlth supporT in Atrial Fibrillation Self-Management (CHAT-AF-S): rationale and randomised controlled trial design' was submitted for review. Overall, this is a well designed study that will be an important contribution to current literature in this area.	Thank you for your positive feedback. We appreciate your thoughtful comments, which we have addressed below.
Major issues:<br>- Please confirm that this study is still ongoing, as it would not be appropriate to publish a protocol on a study that has been completed.	We confirm that while recruitment has finished, the study is still ongoing as delays in finalising data collection and patient follow-up procedures lasting up to May 2026 have delayed database lock and analysis beyond what was initially expected. We have modified the text accordingly to reflect that database lock and analysis are now planned for mid 2026:<br>"Database lock and analysis are planned for mid 2026."
- Please comment on the use of AFEQT as a primary outcome measure. How was this determined? Was there any lived experience engagement in the choice or primary outcome measure?	We have added information on the selection of AFEQT as the primary outcome. The choice was informed by its robust validation, clinical relevance, mention in international guidelines (European Society of Cardiology AF guidelines 2024), previous use as primary outcome in published randomised controlled trials (Mark DB et al. Effect of catheter ablation vs medical therapy on quality of life among patients with atrial fibrillation: the CABANA randomized clinical trial. JAMA 2019), and our own pilot study and qualitative analysis of patient perspectives, as described below:<br><br>"Choice of the primary outcome follows international guideline recommendations for assessment of quality-of-life and patient-reported

	outcomes in AF clinical trials[4]; this choice was further guided by our previous pilot trial 'Coordinating Healthcare with Artificial intelligence Technology for patients with Atrial Fibrillation (CHAT-AF)', where we found a significant pre-post improvement in AFEQT overall score in the intervention group[30, 31], and was informed by lived experience (qualitative interviews with pilot participants and co-author investigator living with AF)[32]."
- Are you able to provide further details as to how patient engagement was embedded in to your trial e.g. was a consumer group recruited, how many were recruited, was this through workshops etc)	We have provided further details on how patient engagement was embedded in the trial, elaborating on the co-design process and patient involvement: "Patient perspectives were embedded in CHAT-AF-S through a qualitative evaluation of the pilot CHAT-AF trial, involving semi-structured interviews with 30 participants[33], as well as co-author lived experience. Participants valued the accessible, human-like interactions and continuity of support but emphasised the need for greater personalisation and actionable content, flexible delivery, bite-sized content delivery, and control over the frequency and volume of contact. These findings informed CHAT-AF-S's tailored conversational pathways with options to skip or receive additional information on a given topic during a call, combining voice-call and text-message delivery, choice of time for the automated phone calls, and additional actionable content prompting discussion with a doctor of an individualised AF action plan."<br><br>33.Trivedi R, Shaw T, Sheahen B, Chow CK, Laranjo L. Patient Perspectives on Conversational Artificial Intelligence for Atrial Fibrillation Self-Management: Qualitative Analysis. J Med Internet Res 2025;27:e64325.
- Was there any limitations placed on AF type for inclusion in the study (paroxysmal vs persistent vs permanent)? If so, was any randomisation stratification undertaken for this?	There were no specific limitations placed on AF type (e.g., paroxysmal vs persistent vs permanent), and randomisation was stratified by sex and site, not AF type. However, we will explore heterogeneity of treatment effect by type of AF, as added below:<br>"We will explore heterogeneity of treatment effect by sociodemographic characteristics, CHA2DS2VA score, recency of AF diagnosis, type of AF, and recruitment setting (inpatient versus outpatient); results will be presented displaying stratification of the outcome by subgroup."
- Was inclusion in a current cardiac rehabilitation program a reason for exclusion from the trial?	Inclusion in a current cardiac rehabilitation program was not a specific exclusion criterion, though patients on concurrent active interventional studies focused on AF education were excluded.

- Are you able to provide any commentary on standard of care at the recruiting institutions with respect to education and risk factor management?	We have added information on the standard of care, as follows:<br>"The control group will receive usual care, comprising scheduled visits and clinical advice provided by their cardiologist or primary care doctor in accordance with contemporary Australian clinical guidance[29]. This may include stroke- and bleeding-risk assessment, anticoagulation where indicated, rate- or rhythm-control treatment, management of cardiovascular risk factors and comorbidities, patient education, clinical monitoring, and referral for cardioversion or catheter ablation where clinically appropriate. The specific treatments and frequency of follow-up are determined by the treating clinicians and will not standardised by the trial."
Minor issues:<br>- I would suggest a longer duration for qualitative interviews e.g. 30-60 minutes per participant.	Thank you for this suggestion. While we initially estimated 10 to 30 minutes to minimize participant burden, we are open to allowing interviews to naturally extend up to 60 minutes if participants wish to share more in-depth experiences. We have adjusted the estimated duration accordingly in the manuscript:<br>"we predict a minimum of 20 patients will be interviewed and estimate that each interview will last on average 30 to 40 minutes"

VERSION 2 - REVIEW

**Reviewer**	**1**
**Name**	**Darroudi, Susan**
**Affiliation**	**Mashhad University of Medical Sciences**
**Date**	**19-Jun-2026**
**COI**	

Dear authors

Thank you for your careful consideration. All of my comments have been addressed.

**Reviewer**	**3**
**Name**	**Gallagher, Celine**
**Affiliation**	**Adelaide University, Australian Dysautonomia and Arrhythmia Research Collaborative**
**Date**	**10-Jul-2026**

COI

Thank you for answering my queries. I have nothing further to add.
